# Doctor, when can I drive? – Influence of muscle weakness of dorsal flexors and plantar flexors from the ankle joint on driving ability

**DOI:** 10.1186/s12891-025-09230-6

**Published:** 2025-10-28

**Authors:** Dominique Schoeps, Max Prost, Falk Hilsmann, Felix Lakomek, Erik Schiffner, Pascal Jungbluth, Max Joseph Scheyerer, Joachim Windolf, David Latz

**Affiliations:** Department of Orthopedic and Trauma Surgery, Medical Faculty and University Hospital Düsseldorf, Heinrich-Heine-University Düsseldorf, Moorenstraße 5, Düsseldorf, 40225 Germany

**Keywords:** Driving ability, Ankle muscle weakness, Dorsiflexion, Plantarflexion, Driving simulator, Reaction time

## Abstract

**Purpose:**

Dorsiflexion (DF) and plantarflexion (PF) weakness are among the most commonly observed muscle strength impairments of the lower extremities. This may be due to spinal conditions, peripheral nerve damage, trauma or multiple other reasons. The personally used automobile remains the most commonly used mode of individual transportation in Germany. However, it is unclear whether and to what extent DF or PF weakness affects the ability to drive safely. This study aimed to experimentally assess the impact of DF and PF weakness on driving ability.

**Methods:**

Twenty healthy licensed drivers with an annual mileage of > 5000 km participated in this experimental study. A custom-made orthosis was applied to the right leg to simulate both DF and PF weakness. Participants completed two driving simulator scenarios: an emergency braking maneuver (EBM) and stop-and-go traffic (StGo) under controlled conditions and with different levels of strength impairment (3/5 and < 3/5). Driving performance parameters were recorded and statistically analyzed using SPSS 29.

**Results:**

DF weakness significantly prolonged brake pedal activation during EBM (2238 ms vs. 2046 ms; *p* < 0.02), while PF weakness had no significant effect. In StGo, PF weakness led to significantly more frequent acceleration use (1.4 vs. 1.05; *p* < 0.02) and increased safety distance (30.3 m vs. 24.8 m; *p* < 0.01). DF weakness resulted in more frequent acceleration use, lower speed, and a reduced safety distance (21.9 m vs. 24.8 m; *p* < 0.05).

**Conclusion:**

PF weakness primarily affects fine motor control in StGo, while DF weakness significantly impacts both EBM and StGo. Compensation mechanisms should be further investigated.

## Introduction

Musculoskeletal health is a key component for people´s personal mobility and driving ability [1, 2]. In our society, personal mobility is still primarily determined by car use. Based on 2020 data from the Federal Statistical Office, over 65% of all working people in Germany reported using their car to commute to work [3]. Thus, the loss of driving ability can result in inability to reach work. This can ultimately lead to significant socioeconomic consequences such as job loss or unemployment. In addition to the problem of getting to work, there is also data that shows that, particularly in regions without well-developed public transport, the inability to use a car is associated with significant restrictions on participation in social life, especially for older people [4, 5]. There is also older data showing that people living in a household without access to a car may have increased mortality in the long term [6]. 

Safe driving requires sufficient joint mobility, strength, and coordination [7–10]. Acceleration and braking, as well as fine-tuning of speed, primarily require movement patterns of the lower extremities [8, 10]. Previous studies suggest that a certain range of motion, particularly in plantar flexion (PF) and dorsiflexion (DF) of the ankle is critical for performing these important maneuvers [8]. However, there is a lack of evidence concerning how much individual strength in preforming PF and DF is needed to drive a car safely.

Musculoskeletal disorders can come along with different kinds of paralysis or neurologic deficits which can affect muscle strength. An example for this might be herniated discs with an affection of nerve roots. Especially herniated discs of L5/S1 can come along with a paralysis of DF or PF from the ankle joint [11–13]. Other musculoskeletal disorders which can affect muscle strength might be traumatic or atraumatic lesion of the peroneal nerve or other traumatic nerve or muscle lesions as well as peripheral neuropathies (e.g. diabetic neuropathy) and systemic neuromuscular disorders (e.g., myopathies, motor neuron disease) [14].

It is already known that surgery for a herniated disc in patients with radiculopathy or preoperative paresis leads to an improvement in reaction time when driving compared to the immediate preoperative status. It is already known that surgery for disc herniation in patients with radiculopathy or preoperative paresis leads to an improvement of driving reaction time compared with the immediate preoperative status [15–17].

However, it is not yet known up to what level of strength driving is safe, or whether there is a limit value in relation to the manual muscle strength grading system beyond which it is no longer safe to drive a car [18, 19]. Furthermore, current literature lacks data on the ability to fine-tune the acceleration process in patients with weakness of the dorsiflexors or plantar flexors.

Knowledge about this would be very important to give recommendation concerning driving capability in patients with these functional disorders. Depending on which limitations of driving ability can be identified in patients with the various forms of muscular weakness, it must be evaluated in the future whether temporary or permanent driving bans or individual examinations of driving ability might be necessary for these patients.

The aim of this study was therefore to investigate whether muscular weakness with reduced DF or PF strength from the ankle joint leads to changes in driving ability. To investigate this, changes in braking behavior and fine-tuning of the acceleration process during driving were analyzed.

## Material and methods

This is an experimental study. A positive approval by the ethics committee (local ethics committee of the Medical Faculty from University Hospital Düsseldorf) was obtained prior its initiation (2021 − 1336). Prior the procedure, an informed consent was obtained, and each participant completed a standardized questionnaire [20]. Only healthy volunteers possessing a driver’s licence and driving their own car at least 5000 km per year the last three years were included. There is no clearly defined threshold for annual mileage that qualifies someone as a seasoned driver. Many car insurance companies consider a driver to be a seasoned driver if they drive more than 5,000–6,000 km annually. Therefore, this study also set a threshold of more than 5,000 km of annual mileage as an inclusion criterion. Volunteers with known injuries of the lower limb, neurological disorders or signs of paralysis were excluded from the study.

### Simulation of paresis

Initially, before the orthosis was applied, each subject was manually examined by each of the surgeons so that they could get an impression of the existing baseline strength level.

**Fig. 1:**
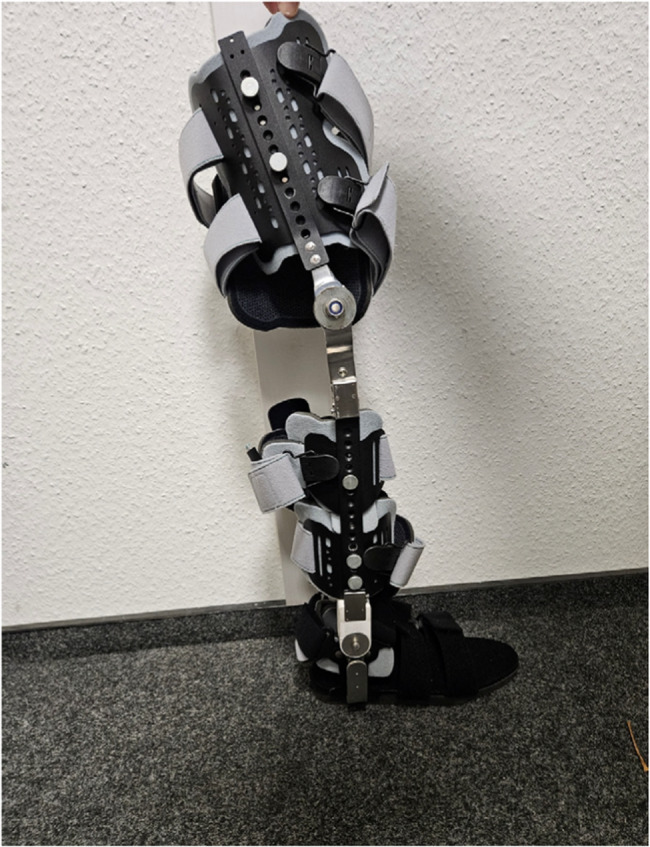
Customized orthosis from Koppetsch Company, that can be easily adjusted, a restriction of movement in the knee or ankle joint can be performed by fully or partial blocking the joints with a torch blocking wrench

Then a customized cross-knee joint orthosis (*see* Fig. 1) was applied to the right leg and attached with velcro fasteners. This orthosis has a joint that can be individually adjusted. The orthosis is a custom-made product that has not been registered or externally validated.

Within the scope of the experiment, the driving ability was examined and in this context the degrees of strength of the ankle joint or the impairment of the ankle joint while driving a car were investigated. The joint can be adjusted to different degrees of force. The force levels investigated were < 3/5, 3/5 and 5/5 (control) according to P. Kendall and E. K. McCreary “Muscles, Testing and Function” [19]. The applied force levels are explained again in Table 1. The resistance of the adjustable joint was created with a torque wrench by the principal investigator. After applying the appropriate resistance, it was checked manually by 3 independent orthopedic and trauma surgeons each with more than 10 years of experience whether a DF or a PF could be performed from a neutral position according to the desired force level (3/5 or < 3/5). The resistance was readjusted by the principal investigator until all 3 agreed with the applied forced for the different levels of power.

**Table 1:** Explanation of the applied force levels according to P. Kendall and E. K. McCreary “Muscles, testing and function”[19]

Force Level	Explanation
5/5	Full strength, no restriction (control)
3/5	Movement against gravity possible, but not against resistance
< 3/5	Movement against gravity not possible

### Driving simulation

All participants were seated in a uniform and standardized position in a driving simulator (Typ Trainer; Foerst Fahrsimulatoren GmbH, Wiehl, NRW, Germany). The participants initially adopted a sitting position that remained constant between the different scenarios. Care was taken to ensure an adequate distance from the pedals, so that the leg was bent approximately 30–45° at the knee and the heel was always firmly in contact with the ground. Furthermore, a distance of approximately 25–30 cm from the steering wheel was set. This type of driving simulator has already been used in previous scientific studies [21]. After getting familiar to the driving simulator in a three-minute free driving scenario, each participant completed the driving courses 5 times: (a) without any movement restrictions (control); (b) with a simulated 3/5 paresis for the dorsiflexors; (c) with a simulated < 3/5 paresis for the dorsiflexors; (d) with a simulated 3/5 paresis for the plantar flexion and (e) with a simulated < 3/5 paresis for the plantar flexion. A randomized order for a) - e) for each participant was chosen to minimize learning effects. Every participant was instructed to drive as fast but also as accurately as possible. There were no planned breaks between the individual scenarios and the various strength level restrictions. Adjustments to the orthosis were made if necessary, otherwise the driver immediately continued with the next ride in the driving simulator. The following two scenarios were part of the driving course.

#### Follow car scenario

This scenario was developed to analyze the impact on fine motor skills required in stop-and-go traffic. During the first simulation, the participant had to follow another car in front of them, with the goal of keeping pace with the other car while maintaining a self-selected, consistent distance. The simulator automatically recorded the speed of the car (in kilometers per hour), the number of brake and acceleration hits as well as the distance to the car ahead (in meter) during the entire course. This route followed a special custom-made sequence:

- Initial phase:
  - Car in front starting with 30 km/h and remains at 30 km/h for 15 s.
  - Participant creating a self-chosen distance to the car in front.
- Phase 1:
  - Rapid acceleration to 50 km/h.
  - Remain at 50 km/h for 10 s.
  - Slow deceleration to 30 km/h.
- Phase 2.
  - Remain 30 km/h for 10 s.
  - Rapid deceleration at 10 km/h.
  - Remain 10 km/h for 10 s.

#### Emergency brake scenario

This scenario was developed to analyze the impact of emergency braking skills. The participant was driving along a lightly traffic-free street at a speed of 70 km/h. The participant was instructed to drive straight ahead. Without warning, a “STOP” sign appeared on the display, prompting the participant to perform an emergency braking maneuver. Meanwhile any information concerning the use of the brake, the time (in milliseconds) until the car reached 0 km/h and the reaction time (in milliseconds) the participants need to change from speed to brake paddle was recorded. To analyze the different biomechanical effects, the emergency braking scenario was evaluated in two phases: Phase 1: No further accelerator pedal operation, but the brake is not yet applied; Phase 2: The brake is applied until a standstill (speed = 0 km/h). Initial reaction time was not included in the final analysis, as we believe it is independent of the reduction in strength and depends more on other personal factors. To keep the results clear and unambiguous, these measurements were not included.

### Data cleaning

The data provided by the Foerst Simulator were first visualized, and any artifacts were identified and excluded using a custom-made program.

### Statistical analysis

Statistical analyses were conducted using Excel^®^ (Microsoft Corporation, Redmond, Washington, United States) and IBM SPSS Statistics version 29 (version 29.0.2; IBM Corp., Armonk, NY, USA). Linear mixed-effects models with repeated measures were applied. The model included condition as a fixed effect and subject as a repeated factor. Restricted maximum likelihood estimation (REML) was used, and the covariance structure for repeated measures was specified as unstructured.

### Model outcomes and comparisons

Estimated marginal means for each condition were computed. Pairwise contrasts with LSD adjustments for multiple comparisons were conducted to compare conditions.

### Range of motion (ROM) data

Although joint range of motion (ROM) parameters were calculated for each condition (unrestricted, restricted with strength grades < 3/5 and 3/5), these data were used primarily for internal consistency checks and were not included in the current analysis, as detailed evaluation of ROM patterns and compensatory mechanisms is beyond the scope of this manuscript.

### Post hoc testing

Additional pairwise post hoc comparisons were performed based on the estimated marginal means to examine differences between groups (unrestricted, restricted with strength grades < 3/5 and 3/5) and maneuvers (follow car/emergency brake). A significance threshold of *p* < 0.05 was applied.

### Sample size

Regarding the necessary sample size, advice and calculations were carried out by an external statistician.

## Results

The researchers included 20 healthy volunteers in this investigation. The participants had a mean age of 30.2 (SD ± 4.45) years. 13 were male and 7 were female. The average body height of the participants was 181.8 cm **(**SD ± 10,73). The average body weight of the participants was 75.71 kg (SD ± 12,14).

### Follow car scenario

The individual parameters were measured for each subject for possible movement restrictions (DF, PF) at < 3/5 and 3/5 in the different phases 1 and phase 2. The minimum, maximum and mean speed was specified here (Table 2 und 3), as well as the brake application and the frequency of brake and acceleration hits. In addition, the minimum, maximum and mean distance to the vehicle in front are specified for each of the above-mentioned variations.

**Table 2:** Results from the follow car scenario with restrictions of the plantar flexion in the ankle joint

		Control	3/5 (mean/SD)	*p*-level	< 3/5 (mean/SD)	*p*-level
Phase 1	maximum speed (km/h)	54,45	53,4/2,90	0,27	53,49/3,49	0,32
	minimum speed in km/h	28,30	28,95/1,61	0,34	28,68/1,79	0,54
	mean speed in km/h	40,75	40,08/1,74	0,15	40,00/2,03	0,15
	acceleration hits	1,05	1,4/0,60	**0**,02*	1,25/0,79	0,30
	maxmium distance to car in front (m)	31,74	35,94/10,64	**0**,05*	35,58/9,68	0,14
	minimum distance to car in front (m)	19,66	22,03/8,40	0,09	21,02/6,55	0,40
	mean distance to car in front (m)	25,84	28,75/9,11	0,07	28,17/7,82	0,27
Phase 2	maximum speed (km/h)	52,35	52,15/3,19	0,84	52,7/3,84	0,72
	minimum speed in km/h	22,34	23,24/5,44	0,53	23,14/4,53	0,58
	mean speed in km/h	35,96	35,97/2,44	0,98	35,70/2,04	0,56
	brake hits	1,40	1,05/0,22	0,11	1,25/0,55	0,55
	Acceleration hits	2,50	2,5/0,83	1,00	2,25/0,55	0,17
	maxmium distance to car in front (m)	30,48	35,87/12,03	**0**,04*	35,41/10,81	0,08
	minimum distance to car in front (m)	19,81	24,64/11,76	**0**,02*	24,25/10,27	0,08
	mean distance to car in front (m)	24,77	30,33/11,39	**0**,01*	29,51/11,15	0,08

**Table 3:** Results from the follow car scenario with restrictions of the dorsiflexors in the ankle joint

		Control	3/5 (mean/SD)	*p*-level	< 3/5 (mean/SD)	*p*-level
Phase 1	maximum speed (km/h)	54,45	53,94/3,18	0,56	53,80/3,74	0,46
	minimum speed in km/h	28,30	28,345/2,10	0,94	27,57/2,53	0,40
	mean speed in km/h	40,75	41,04/1,72	0,41	39,9/1,67	**0**,05*
	acceleration hits	1,05	1,65/1,04	**0**,02*	1,65/1,35	0,07
	maxmium distance to car in front (m)	31,74	27,22/6,64	**0**,05*	31,22/8,42	0,83
	minimum distance to car in front (m)	19,66	16,56/4,75	0,07	17,69/6,15	0,25
	mean distance to car in front (m)	25,84	21,99/5,36	**0**,05*	25,03/7,21	0,68
Phase 2	maximum speed (km/h)	52,35	51,74/2,31	0,50	53,65/4,27	0,38
	minimum speed in km/h	22,34	22,00/5,77	0,80	23,32/5,73	0,52
	mean speed in km/h	35,96	35,34/1,5	0,15	36,51/1,78	0,25
	brake hits	1,40	1,3/0,57	0,67	1,4/0,99	1,00
	Acceleration hits	2,50	2,8/1,06	0,33	2,6/1,05	0,71
	maxmium distance to car in front (m)	30,48	26,49/6,41	0,06	30,47/8,96	1,00
	minimum distance to car in front (m)	19,81	17,65/5,78	0,24	19,55/8,07	0,90
	mean distance to car in front (m)	24,77	22,245/6,07	0,17	24,45/8,08	0,88

For the DF there was a significantly lower mean speed in phase 1 with a simulated paresis of 3/5 compared to the control group (39.9 vs. 40.75; *p* < 0.05). Further, when a paresis of 3/5 was simulated, there were significant higher acceleration hits (1.65 vs. 1.05; *p* < 0.02) and a significant lower mean and maximum distance to the car in front (21.99 vs. 25.84; *p* < 0.05/27.22 vs. 31.74; *p* < 0.05) in phase 1 compared with the control scenario. See also Table 3 for the complete results for the restrictions in DF.

Tables 2 and 3 present a detailed comparison of the outcome measures for each experimental group, stratified by restriction levels (3/5 and < 3/5), in relation to the control group. Specifically, Table 2 reports the results for PF and Table 3 displays the corresponding values for DF. Both tables refer to performance during the *follow car maneuver*, providing insight into how varying degrees of restriction impact driving behaviour compared to the control condition.

For the PF there was a significantly higher number of acceleration hits in phase 1 with a simulated paresis of 3/5 compared to the control group. (1.4 vs. 1.05; *p* < 0.02). Further, in phase 1, the maximum distance to the car in front was significantly higher (35.94 vs. 31.74; *p* < 0.05) when a paresis of 3/5 was simulated compared to the control scenario. In phase 2 again there were significant changes in the maximum distance to the car in front (35.87 vs. 30.48; *p* < 0.04) as well as for the minimum distance (24.64 vs.19.81; *p* < 0.02) and the mean distance (30.33 vs. 24.77; *p* < 0.01). See also Table 2 for the complete results for the restrictions in PF.

###  Emergency brake scenario

Here, the time is given in milliseconds in each case. The individual parameters were measured for each subject for possible movement restrictions (DF, PF) at < 3/5 and 3/5 in the different phases 1 and 2 compared to the control group (Tables 4 and 5).

For the PF there were no significant changes in performing an emergency braking. See also Table 4 for the complete results for the restrictions in PF.

For the DF there were no significant changes in phase 1 (no further accelerator pedal operation, but the brake has not yet been applied either) but there were in phase 2 (brake is applied until full stop) of the scenario. The time until full stop was significantly longer in both restricted groups when compared to control. See also Table 5 for the complete results for the restrictions in DF.

**Table 4:** Results from the emergency braking scenario with restrictions of the plantar flexion in the ankle joint

	Control Mean/SD	3/5 Mean/SD	*p*-level	< 3/5 Mean/SD	*p*-level
Phase 1 Time (ms)	181,90/101,17	170,2/94,50	0,71	183,50/138,34	0,96
Phase 2 Time (ms)	2046,40/269,70	2061,25/304,03	0,78	2139,60/393,17	0,29

**Table 5:** Results from the emergency braking scenario with restrictions of the dorsiflexors in the ankle joint

	Control Mean/SD	3/5 Mean/SD	*p*-level	< 3/5 Mean/SD	*p*-level
Phase 1 Time (ms)	181,90/101,17	208,40/236,96	0,47	195,15/141,84	0,72
Phase 2 Time (ms)	2046,40/269,70	2221,40/420,09	**0**,05*	2238,00/408,65	**0**,02*

## Discussion

In this investigation it was analyzed whether muscular weakness with reduced force of the DF or PF in the right ankle joint leads to changes in the ability of driving a car. The results showed that weakness in both DF and the PF affects the driving ability.

According to our results a limitation in the PF primarily affects motor control during precision tasks, such as in the follow-car scenario, whereas a limitation in the DF affects both motor control during precision tasks and gross motor skills, such as in the emergency braking scenario. The results of this study are consistent with the available literature, which provides evidence that lower extremity motor deficits affect driving ability[15–17, 22, 23]. In addition, these findings offer detailed insights into the extent of this influence. The performed follow car scenario especially analyzed motor control during precision tasks like the fine adjustment of the accelerator. The analysis revealed that reduced force in both DF and PF was significantly associated with an increase number of acceleration hits (see Tables 2 and 3).

This could be explained by the fact that participants were unable to adjust driving speed by small changes in the position of the right ankle joint. Instead, when a reduced force of DF or PF was simulated, they had to fully depress or completely release the accelerator pedal to adjust speed. Both restrictions also resulted in significant increase in the selected safety distance compared to the control drive.

These changes are might be due to the participants’ feelings of insecurity caused by the reduced force. Previous studies have shown that the selected safety distance is significantly influenced by psychological factors such as feelings of insecurity or inattention [24, 25]. However, since no psychological measurements were taken in this study, this explanation remains a hypothesis that should be clarified with specific measurements in the future investigations.

The significantly reduced driving speed in participants with simulated PF weakness is probably also explained by these psychological factors.

Thaler et al. (2012) already demonstrated that paresis of the lower limbs can significantly impact the emergency braking performance [16]. However, their results had some limitations: they did not differentiate between different types or degrees of paresis and did not break down the braking process into separate stages. In contrast, our findings provide a more detailed analysis. Furthermore, our results indicate that especially reduced force of the DE in the right ankle joint has an influence on the emergency braking. We observed that reduced strength of the dorsiflexors (DE) in the right ankle joint influenced braking performance primarily during the application of the brake pedal (phase 2), but not significantly during the transition from the accelerator to the brake pedal (phase 1). One possible explanation for this is that while the transition phase requires movement speed and coordination, the actual braking force in phase 2 depends more on stabilising the ankle and performing controlled plantar flexion from a dorsiflexed position. This suggests that insufficient DE strength may impair the ability to maintain optimal foot positioning and apply consistent force during braking. Future studies could examine this phase-specific relationship in more detail using motion capture and muscle activation analysis, for example.

In contrast, no significant changes in emergency braking performance were observed when plantar flexor (PF) strength was reduced. This is consistent with the findings of Latz et al. (2020), who reported that, during braking, plantar flexion from a dorsiflexed position is utilised, but this may be supported by compensatory mechanisms in adjacent joints. For instance, limitations in ankle motion could be at least partially offset by an increased range of motion in the knee and/or hip, as well as greater activation of the quadriceps and gluteal muscles. These compensatory strategies may explain why reduced PF strength alone did not measurably impact on braking performance in our sample.

Noticeable is that a reduced force of < 3/5 otherwise than a reduced force of 3/5 mostly does not lead to significant changes in the driving performance.

Compensation mechanisms could again be the reason for these results. Especially in cases of severe limitations, it may be possible that the body resorts to existing compensation mechanisms earlier than in cases of milder limitations. But, due to a lack of evidence according this topic, this hypothesis requires further investigation. Increased safety distance and reduced driving speed observed in our study likely represent protective compensatory strategies that may reduce collision risk by increasing the margin for stopping and reaction time. However, these behavioural adaptations may also indicate reduced driver confidence and could led to hidden costs, such as increased cognitive load, fatigue, or excessive use of proximal joints. Therefore, while these changes appear beneficial in controlled settings, their overall impact on real-world driving safety remains to be fully determined. Future studies should investigate these dynamics longitudinally and in naturalistic driving conditions over time to better understand the functional significance of such compensations.

Such compensation mechanisms are already described for the upper extremity [21].Comparable compensation mechanisms for the lower extremities when driving a car are to be expected, especially since these have already been described in part for walking in patients with unilateral calf muscle weakness. There, Waterval et al. (2018)describe an increased range of motion in the hip of the affected side as well as in the contralateral leg compared to an unaffected control group[10, 26]. There is still a significant range of motion available in the knee joint beyond the normally used range of motion. By increasing the range of motion used in the knee joint, the reduced strength or mobility in the ankle joint can potentially be compensated for.

Further studies should therefore address the question of whether muscular weakness in knee flexion and extension in the right knee also has an impact on driving ability. In addition, other compensatory mechanisms for muscle strength loss in the lower extremities during driving should be analyzed. Motion capture systems could be used to analyze the precise influence of various forms of strength loss in the lower extremities on the altered range of motion of other joints. Likewise, in a next step, another study could examine how driving behavior changes in patients with known paresis or muscle weakness without having to simulate these.

The absence of significant changes in performance despite muscular weakness suggests that compensation mechanisms may be in operation. These mechanisms may be employed earlier and more effectively in cases of severe limitations than in cases of milder impairments. However, due to the limited evidence available, this hypothesis requires further investigation.

Such mechanisms have been described in the upper extremity, and comparable adaptations in the lower extremities in driving scenarios are plausible. For example, Waterval et al. (2018) reported increased hip range of motion on the affected side and in the contralateral leg during walking in patients with unilateral calf muscle weakness, compared to an unaffected control group. The knee joint also has a considerable reserve range of motion reserve beyond what is typically used, which allows potential compensation of reduced ankle strength or mobility.

Future studies should therefore investigate whether muscular weakness in knee flexion and extension of the right knee also affects driving ability. Additionally, other compensatory strategies for muscle strength loss in the lower limbs during driving should be analysed. Motion capture systems could help to quantify how different forms of lower-limb strength loss alter the range of motion in other joints. A next step could be to examine how driving behaviour changes in individuals with documented paresis or muscle weakness without relying solely on simulation.

Overall, this study adds new evidence-based functional biomechanical parameters that can help to evaluate driving fitness.

The findings of this study may contribute valuable insights for assessing driving fitness, particularly in individuals with neuromuscular or orthopedic handicaps affecting the lower extremities. Although this investigation was conducted on healthy participants with simulated muscular weakness, the observed biomechanical alterations provide a foundation for understanding real-world significance in medically impaired persons, such as individuals with paresis following stroke, multiple sclerosis, peripheral nerve injury, or orthopedic conditions such as ankle arthrosis (Waterval et al., 2018) [26].

Driving fitness in such populations is typically evaluated by medical professionals, including rehabilitation physicians, neurologists, or occupational therapists, often in collaboration with certified driving assessment specialists (Korner-Bitensky et al., 2009) [27]. The biomechanical parameters identified in this study—such as altered emergency braking performance and reduced fine motor control of the ankle—could inform the development or refinement of clinical screening tools.

In particular, motion-based evaluations or driving simulator assessments focusing on fine and gross motor control of the right lower extremity could be considered in clinical practice. For instance, tests assessing the ability to perform controlled ankle dorsiflexion or plantarflexion under time constraints may help identify individuals at risk of impaired driving performance [28]. Moreover, motion capture systems or instrumented pedals in simulators could provide objective data to support return-to-driving decisions or identify the need for vehicle adaptations [7, 8].

Further research is necessary to reinforce the understanding of driving fitness under conditions of musculoskeletal impairment. Future studies should not only examine the direct effects of specific movement limitations on driving performance, but also explore the compensatory mechanisms of adjacent joints that may reduce these impairments. For example, an increased range of motion or muscle activation in the knee or hip could potentially compensate for restricted ankle mobility or reduced muscle strength. Identifying which compensatory strategies are most effective could allow for a more accurate evaluation of driving fitness—one that does not simply consider the presence of a limitation, but also the functional ability to compensate for it [7, 26].

This investigation has some limitations that need to be mentioned. When evaluating driving fitness, different car types must be taken in account. In this investigation it was decided to perform the maneuvers with automatic transmissions. This was done because only the limitations of the right leg should be analyzed and these should not be distorted by movements of the left leg during the shifting procedure. Further it must be mentioned that in this investigation only healthy participants with simulated restrictions have been analyzed, rather than patient with actual neuromuscular impairments. This is important for interpreting external validity, as patient with real impairments may present with greater variability in severity and may have developed compensatory mechanisms over time. Thus, the collective of participant is regarded as a homogeneous study population, which limits the direct applicability of the findings to clinical populations.

Another possible limitation of the study is that the restrictions applied were completely new to the subjects, whereas in patients who have been suffering from such restrictions for a long time, certain adaptation processes may have taken place.

Additionally, the relatively small sample size (*n* = 20) may limit the study’s generalizability, potentially reducing the statistical power and reliability of the conclusions.

This line of research could eventually lead to a more individualized approach to driving assessments. In cases where individuals demonstrate strong compensatory capacity despite significant local impairments, safe driving may still be feasible.

Accordingly, future investigations should aim to characterize these mechanisms more precisely and determine under which circumstances they may justify a positive assessment of driving capability, even in the presence of significant physical disability.

## Conclusion

Restrictions in DF and PF of the right ankle joint both have a significant impact on driving performance. Limitations of PF primarily affect motor control during precision tasks, as in the follow-car scenario, whereas limitations of DF affect both motor control during precision tasks and gross motor skills, as in the emergency braking scenario. Future studies should include a detailed analysis of joint range of motion and compensatory mechanisms in adjacent joints to further investigate the biomechanical adaptations associated with different strength conditions.

## Data Availability

The datasets generated during and/or analyzed during the current study are not publicly available due to data protection but are available from the corresponding author on reasonable request. The authors are accountable for all aspects of the work (if applied, including full data access, integrity of the data and the accuracy of the data analysis) in ensuring that questions related to the accuracy or integrity of any part of the work are appropriately investigated and resolved.

## Abbreviations

PF: Plantarflexion
DF: Dorsiflexion
